# Supplementary material for: PP1 phosphatase controls both daughter cell formation and amylopectin levels in Toxoplasma gondii
Source: PLoS Biol. 2024 Sep 10;22(9):e3002791. doi: 10.1371/journal.pbio.3002791 (PMC11414933; doi:10.1371/journal.pbio.3002791)
Supplement: S1 Raw Images — (PDF) [file pbio.3002791.s005.pdf]

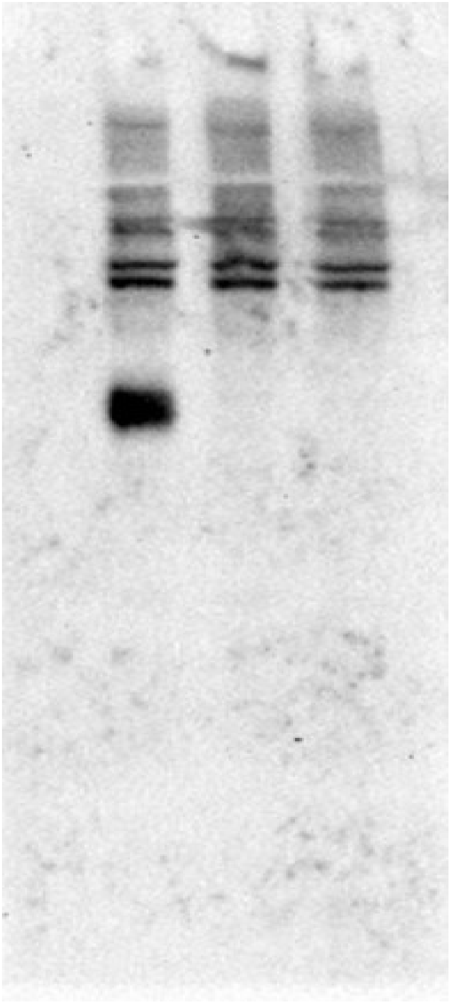

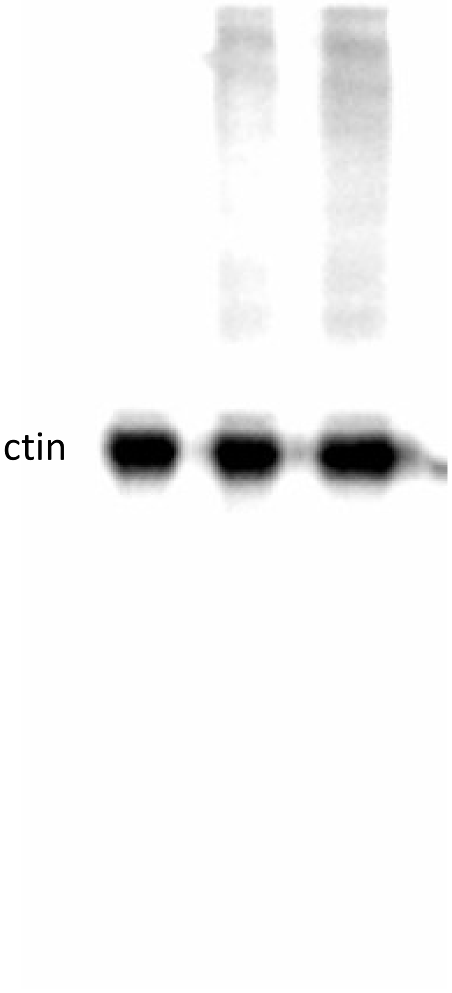

Figure 1c uncropped version

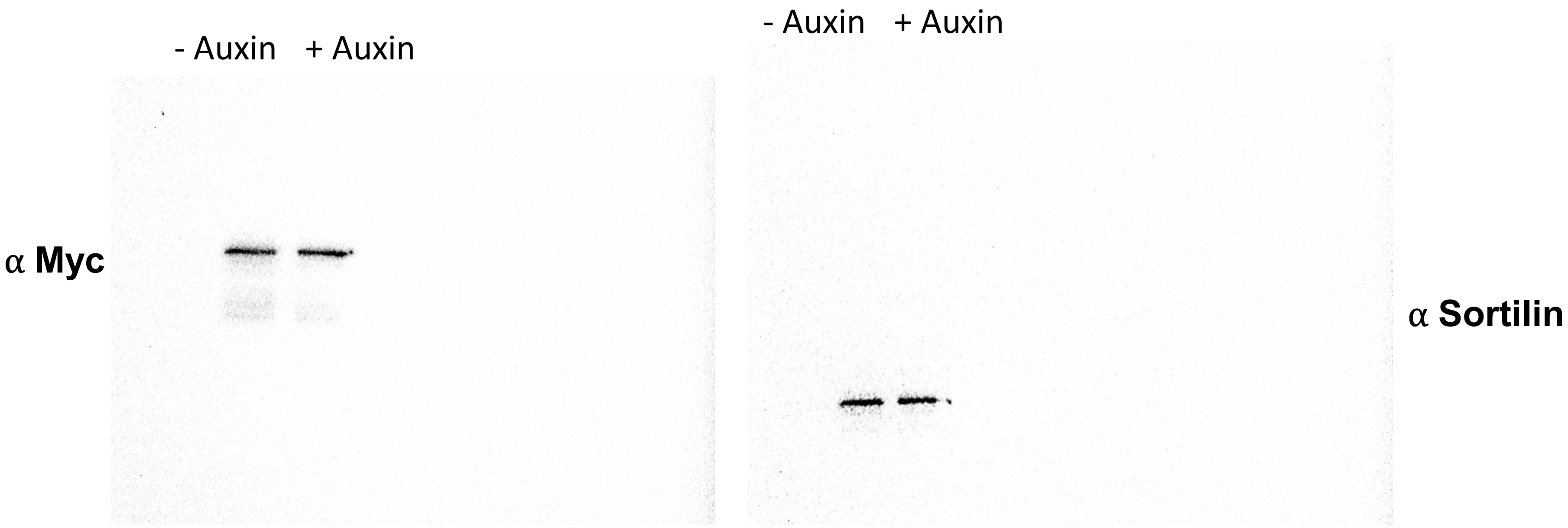

Figure 5f uncropped version

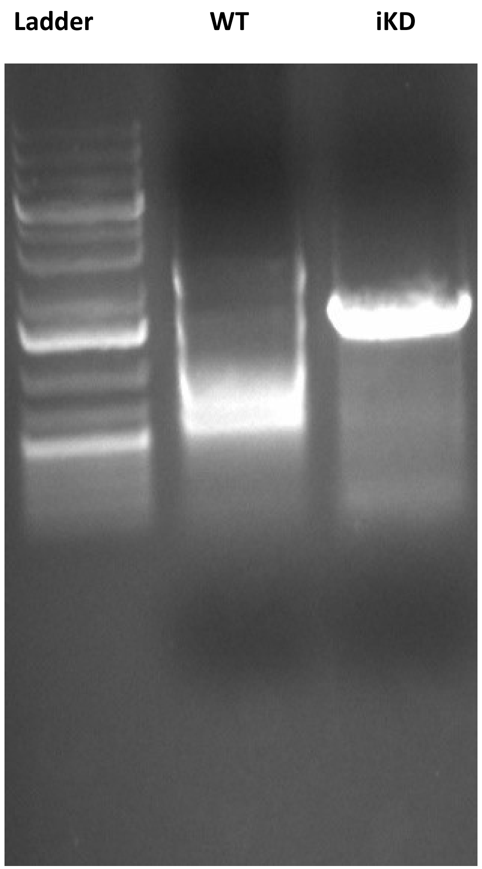

Figure S1a uncropped version
