# Supplementary material for: PP1 phosphatase controls both daughter cell formation and amylopectin levels in Toxoplasma gondii
Source: PLoS Biol. 2024 Sep 10;22(9):e3002791. doi: 10.1371/journal.pbio.3002791 (PMC11414933; doi:10.1371/journal.pbio.3002791)
Supplement: S1 Fig — (a) PCR verifying integration of the HXGPRT-2TA-AID-Ty cassette at the correct genome locus of the iKD TgPP1 mutant. A band corresponding to 1536 using iKD TgPP1 genomic DNA confirms cassette integration compared to using WT genomic DNA. (b) Growth assay of the Parental Tir1 and iKD TgPP1 mutant strains in the absence and presence of auxin treatment for 24 h. A Student’s t test was performed, ns > 0.05, **p < 0.01; mean ± SD (n = 5). (c) Bar graph indicating plaque size produced by the Parental Tir1 and iKD TgPP1 strain in the presence and absence of auxin. Plaque size was determined by measuring the percentage of lysed surface of the plaque assay. Three independent experiments were carried out. A Student’s t test was performed, **p < 0.01; mean ± SD (n = 3). The data underlying this figure can be found in S1 Data. (PDF) [file pbio.3002791.s006.pdf]

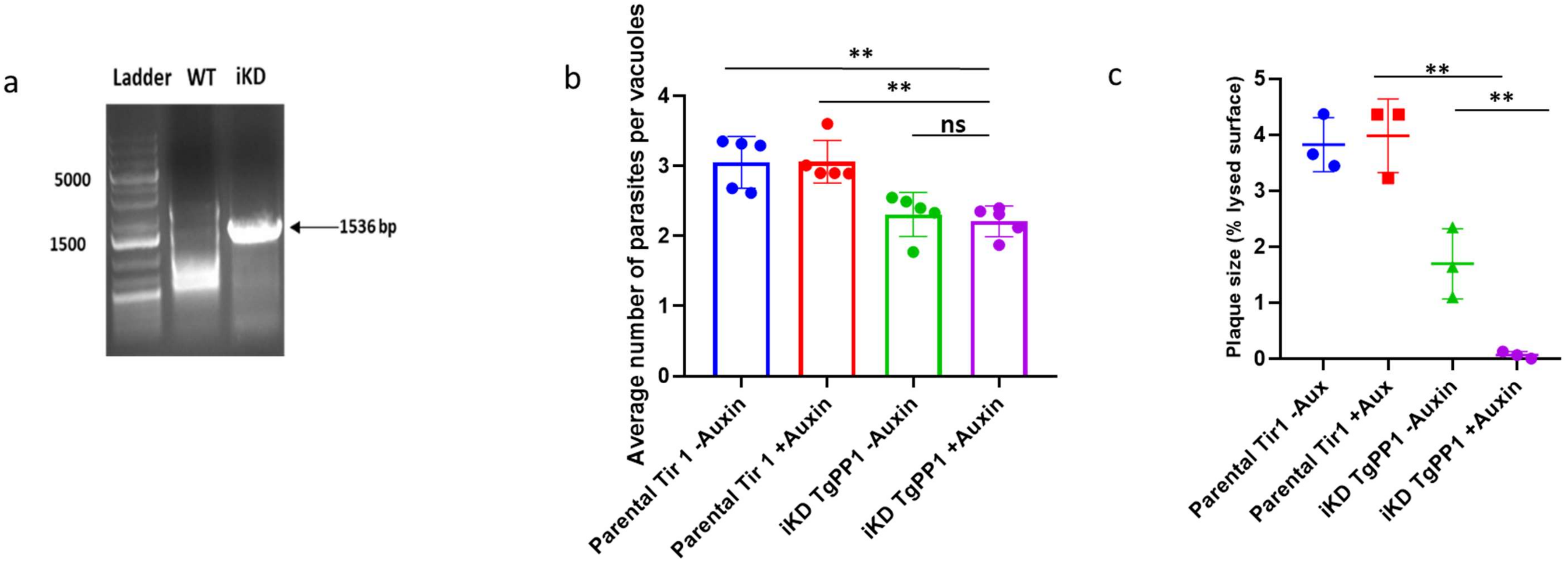

S1 Figure: iKD TgPP1 mutant construction (a) PCR verifying integration of the HXGPRT-2TA-AID-Ty cassette at the correct genome locus of the iKD TgPP1 mutant. A band corresponding to 1536 using iKD TgPP1 genomic DNA confirms cassette integration compared to using WT genomic DNA. **(b)** Growth assay of the Parental Tir1 and iKD TgPP1 mutant strains in the absence and presence of auxin treatment for 24 hours. A Student's *t*-test was performed, ns>0.05, **p<0.01; mean ± s.d. (n=5). **(c)** Bar graph indicating plaque size produced by the Parental Tir1 and iKD TgPP1 strain in the presence and absence of auxin. Plaque size was determined by measuring the percentage of lysed surface of the plaque assay. Three independent experiments were carried out. A Student's *t*-test was performed, **p<0.01; mean ± s.d. (n=3). The data underlying this Figure can be found in S1 Data.
