# Supplementary material for: PP1 phosphatase controls both daughter cell formation and amylopectin levels in Toxoplasma gondii
Source: PLoS Biol. 2024 Sep 10;22(9):e3002791. doi: 10.1371/journal.pbio.3002791 (PMC11414933; doi:10.1371/journal.pbio.3002791)
Supplement: S2 Fig — (a) Confocal imaging of the Parental Tir1 and iKD TgPP1 strains labeled with anti-TgGAP45 (red) in the presence and absence of auxin treatment. DAPI was used to stain the nucleus. Scale bar (1 μm) is indicated in the lower right corner of each individual image. (b) Confocal imaging of the Parental Tir1 and iKD TgPP1 strains labeled with anti-TgEno2 (red) and anti-TgISP1 (green) in the presence and absence of auxin treatment. DAPI was used to stain the nucleus. Scale bar (1 μm) is indicated in the lower right corner of each individual image. (c) Confocal imaging of the Parental Tir1 and iKD TgPP1 strains labeled with TgEno2 (red) and TgIMC1 (green) in the presence and absence of auxin treatment. DAPI was used to stain the nucleus. Scale bar (1 μm) is indicated in the lower right corner of each individual image. (d) Bar graph representing the percentage of Parental Tir1 and iKD TgPP1 vacuoles possessing a collapsed IMC by using anti-TgIMC1 antibodies for labeling the IMC in the absence and presence of auxin treatment for 48 h. A Student’s t test was carried out, ***p < 0.001; mean ± SD (n = 3). (e) Bar graph representing the number of parasite observed by EM with inner membrane defects in the iKD TgPP1 strain in absence of auxin (n = 53), after 24 h of auxin treatment (n = 44) or after 48 h of auxin treatment (n = 46). The data underlying this figure can be found in S1 Data. (PDF) [file pbio.3002791.s007.pdf]

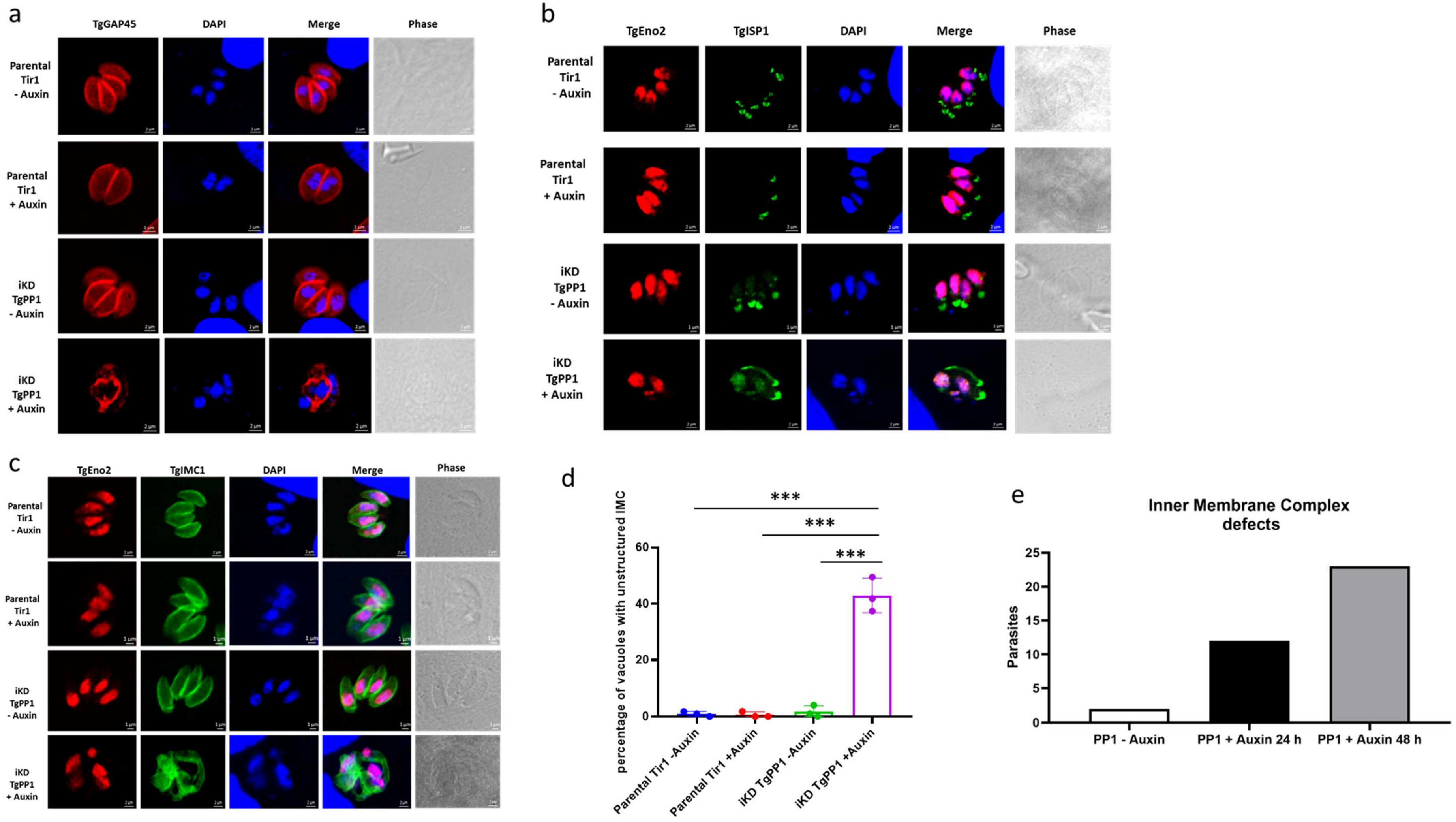

Supplementary Figure 2: iKD TgPP1 demonstrates a collapsed IMC verified through TgGAP45 and TgISP1 labelling (a) Confocal imaging of the Parental Tir1 and iKD TgPP1 strains labelled with anti-TgGAP45 (red) in the presence and absence of auxin treatment. DAPI was used to

stain the nucleus. Scale bar (1µm) is indicated in the lower right corner of each individual image. **(b)** Confocal imaging of the Parental Tir1 and iKD TgPP1 strains labelled with anti-TgEno2 (red) and anti-TgISP1 (green) in the presence and absence of auxin treatment. DAPI was used to stain the nucleus. Scale bar (1µm) is indicated in the lower right corner of each individual image. **(c)** Confocal imaging of the Parental Tir1 and iKD TgPP1 strains labelled with TgEno2 (red) and TgIMC1 (green) in the presence and absence of auxin treatment. DAPI was used to stain the nucleus. Scale bar (1µm) is indicated in the lower right corner of each individual image. **(d)** Bar graph representing the percentage of Parental Tir1 and iKD TgPP1 vacuoles possessing a collapsed Inner Membrane Complex (IMC) by using anti-TgIMC1 antibodies for labelling the IMC in the absence and presence of auxin treatment for 48 hours. A Student's *t*-test was carried out, ***$p<0.001$; mean ± s.d. (n=3). **(e)** Bar graph representing the number of parasite observed by EM with inner membrane defects in the iKD TgPP1 strain in absence of auxin (n=53), after 24h of auxin treatment (n=44) or after 48h of auxin treatment (n=46). The data underlying this Figure can be found in S1 Data.
