# Supplementary material for: PP1 phosphatase controls both daughter cell formation and amylopectin levels in Toxoplasma gondii
Source: PLoS Biol. 2024 Sep 10;22(9):e3002791. doi: 10.1371/journal.pbio.3002791 (PMC11414933; doi:10.1371/journal.pbio.3002791)
Supplement: S3 Fig — (a) Confocal imaging of Parental Tir1 and iKD TgPP1 parasites in the absence and presence of auxin treatment for 48 h labeled with anti-TgCentrin1 antibodies (green). DAPI was used to stain the nucleus. Scale bar (1 μm) is indicated in the lower right corner of each image. (b) Bar graph demonstrating TgCentrin1: nucleus ratio of Parental Tir1 and iKD TgPP1 in the absence and presence of 48-h auxin treatment. A Student’s t test was performed, ns: p > 0.05; mean ± SD (n = 3). For each nucleus, the number of centrin dots is accounted independently from their size. Overall, more than 100 individual nuclei are counted for each biological replicate. (c) Representative expansion microscopy images of the iKD TgPP1 parasites in presence of auxin treatment for 24 h labeled with anti-Nuf2 (kinetochores, red) and anti-acetyl Tubulin (cytoskeleton, green) antibodies. DAPI was used to stain the nucleus. Scale bar (5 μm) is indicated in the lower right corner of each image. (d) Bar graph comparing the number of parasite undergoing Metaphase of Anaphase of the iKD TgPP1 in the absence (n = 414) and presence (n = 426) of 24-h auxin treatment. A Chi2-test was performed, ****: p < 0.001; the number of parasites scored is indicated on top of the graph. The data underlying this figure can be found in S1 Data. (PDF) [file pbio.3002791.s008.pdf]

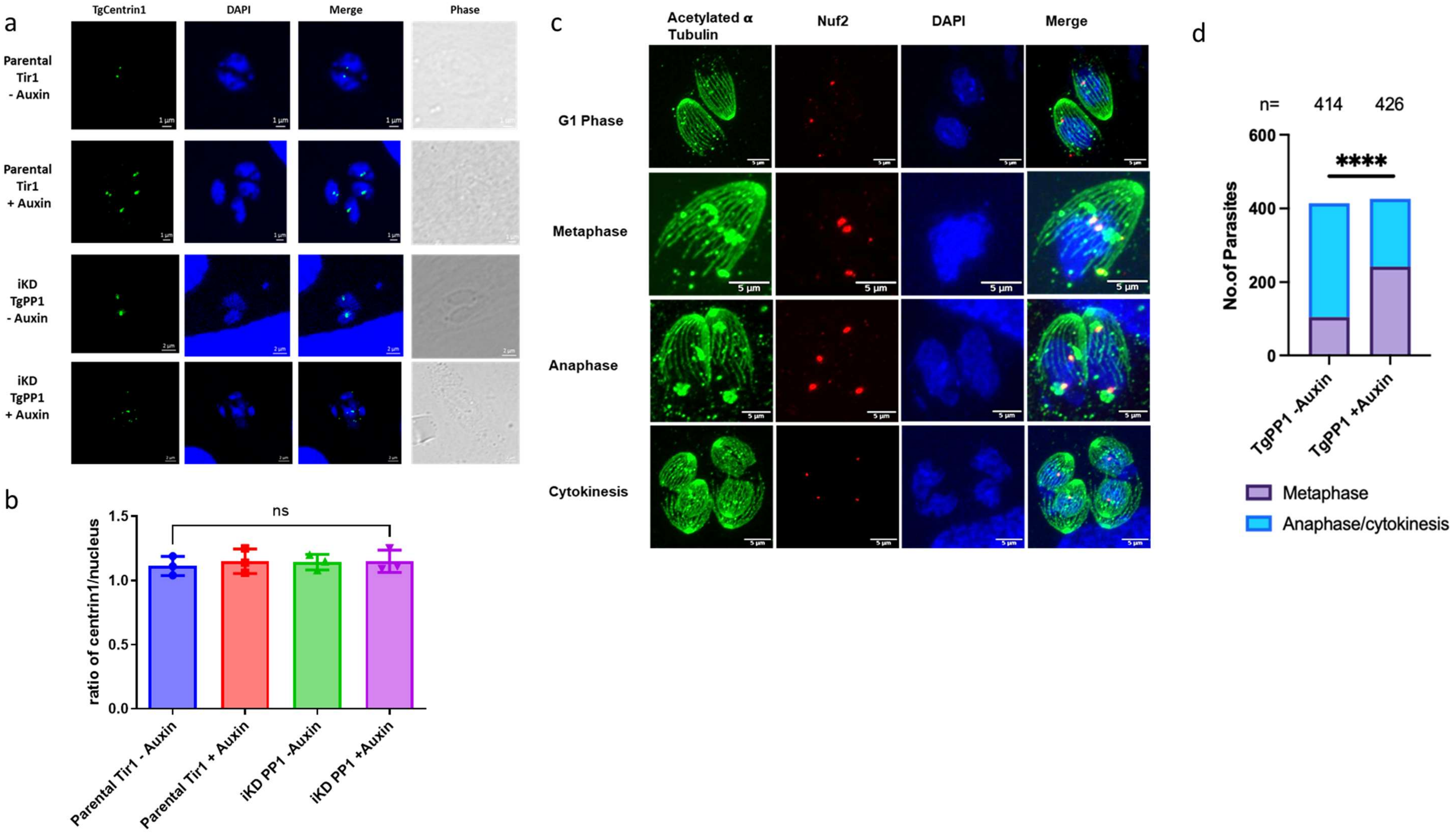

Supplementary Figure 3: Conditional depletion of TgPP1 has a qualitative impact on the outer core centrosome (a) Confocal imaging of Parental Tir1 and iKD TgPP1 parasites in the absence and presence of auxin treatment for 48 hours labelled with anti-TgCentrin1 antibodies

(green). DAPI was used to stain the nucleus. Scale bar (1µm) is indicated in the lower right corner of each image. **(b)** Bar graph demonstrating TgCentrin1: nucleus ratio of Parental Tir1 and iKD TgPP1 in the absence and presence of 48-hour auxin treatment. A Student's *t*-test was performed, ns: $p>0.05$; mean ± s.d. (n=3). For each nucleus, the number of centrin dots is accounted independently from their size. Overall, more than 100 individual nuclei are counted for each biological replicate. **(c)** Representative expansion microscopy images of the iKD TgPP1 parasites in presence of auxin treatment for 24 hours labelled with anti-Nuf2 (kinetochores, red) and anti-acetyl Tubulin (cytoskeleton, green) antibodies. DAPI was used to stain the nucleus. Scale bar (5 µm) is indicated in the lower right corner of each image. **(d)** Bar graph comparing the number of parasite undergoing Metaphase of Anaphase of the iKD TgPP1 in the absence (n=414) and presence (n=426) of 24-hour auxin treatment. A Chi^2-test was performed, ****: $p<0.001$; the number of parasites scored is indicated on top of the graph. The data underlying this Figure can be found in S1 Data.
