# Supplementary material for: PP1 phosphatase controls both daughter cell formation and amylopectin levels in Toxoplasma gondii
Source: PLoS Biol. 2024 Sep 10;22(9):e3002791. doi: 10.1371/journal.pbio.3002791 (PMC11414933; doi:10.1371/journal.pbio.3002791)
Supplement: S4 Fig — (a) Volcano plot demonstrating the differentially expressed genes analyzed from RNA-sequencing of the iKD TgPP1 mutant parasite treated with auxin for 24 h. Differential expression is based on the analysis of 3 biological replicates. Statistically significant differentially expressed genes are indicated in red. However, these do not pass the +/− 1 log2 expression ratio criteria. (b) MA (Bland–Altman) plot demonstrating the differential proteome content in iKD TgPP1 mutant parasites after 24 h of auxin treatment compared to iKD parasite grown in the absence of auxin (control). Statistically significant differentially expressed proteins are indicated in red. The data underlying this figure can be found in S1 Data. (PDF) [file pbio.3002791.s009.pdf]

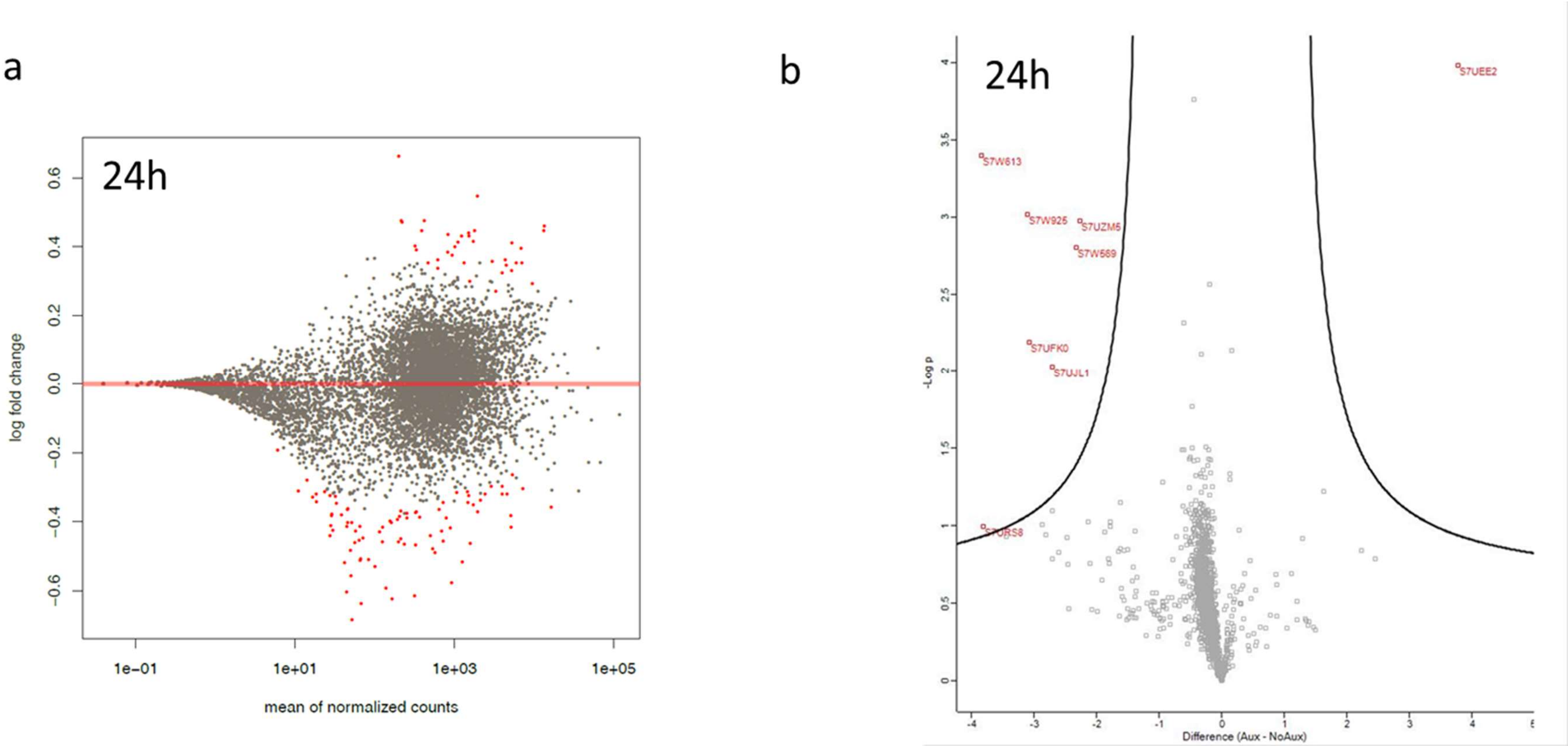

Supplementary Figure 4: RNA-sequencing and proteome analysis at 24 hours of auxin treatment displays only a few differentially regulated genes (a) Volcano plot demonstrating the differentially expressed genes analyzed from RNA-sequencing of the iKD TgPP1 mutant parasite treated with auxin for 24 hours. Differential expression is based on the analysis of three biological replicates. Statistically significant differentially expressed genes are indicated in red. However, these do not pass the +/- 1 log_2 expression ratio criteria. **(b)** MA (Bland-Altman) plot demonstrating the differential proteome content in iKD TgPP1 mutant parasites after 24 hours of auxin treatment compared to iKD parasite grown

in the absence of auxin (control). Statistically significant differentially expressed proteins are indicated in red. The data underlying this Figure can be found in S1 Data.
