# Supplementary material for: PP1 phosphatase controls both daughter cell formation and amylopectin levels in Toxoplasma gondii
Source: PLoS Biol. 2024 Sep 10;22(9):e3002791. doi: 10.1371/journal.pbio.3002791 (PMC11414933; doi:10.1371/journal.pbio.3002791)
Supplement: S7 Fig — (PDF) [file pbio.3002791.s012.pdf]

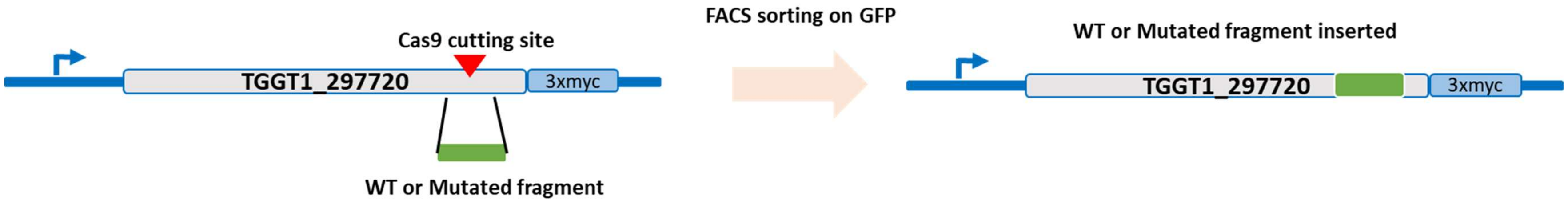

Supplementary Figure 7: Schematic representation of the construction of point mutant of the TGGT1_297720 gene.
