# Supplementary material for: PP1 phosphatase controls both daughter cell formation and amylopectin levels in Toxoplasma gondii
Source: PLoS Biol. 2024 Sep 10;22(9):e3002791. doi: 10.1371/journal.pbio.3002791 (PMC11414933; doi:10.1371/journal.pbio.3002791)
Supplement: S5 Fig — (A) Proportional representation of phosphorylation sites on serine, threonine, and tyrosine for total phosphorylation sites (TOTAL), significant down-regulated phosphorylation sites (DOWN) and significant up-regulated phosphorylation sites (UP). (B) Bioinformatics analysis of potential kinases and/or binding partners based on the surrounding sequence of altered phosphorylation sites revealed the 10 most common kinase and/or binding partner substrate motifs detected among significant phosphoproteins. (C) Sequence logos for significant phosphorylation motifs (left for down-regulated phosphorylation sites, right for up-regulated phosphorylation sites) where the phosphorylated residue (S or T) is centered. (PDF) [file pbio.3002791.s010.pdf]

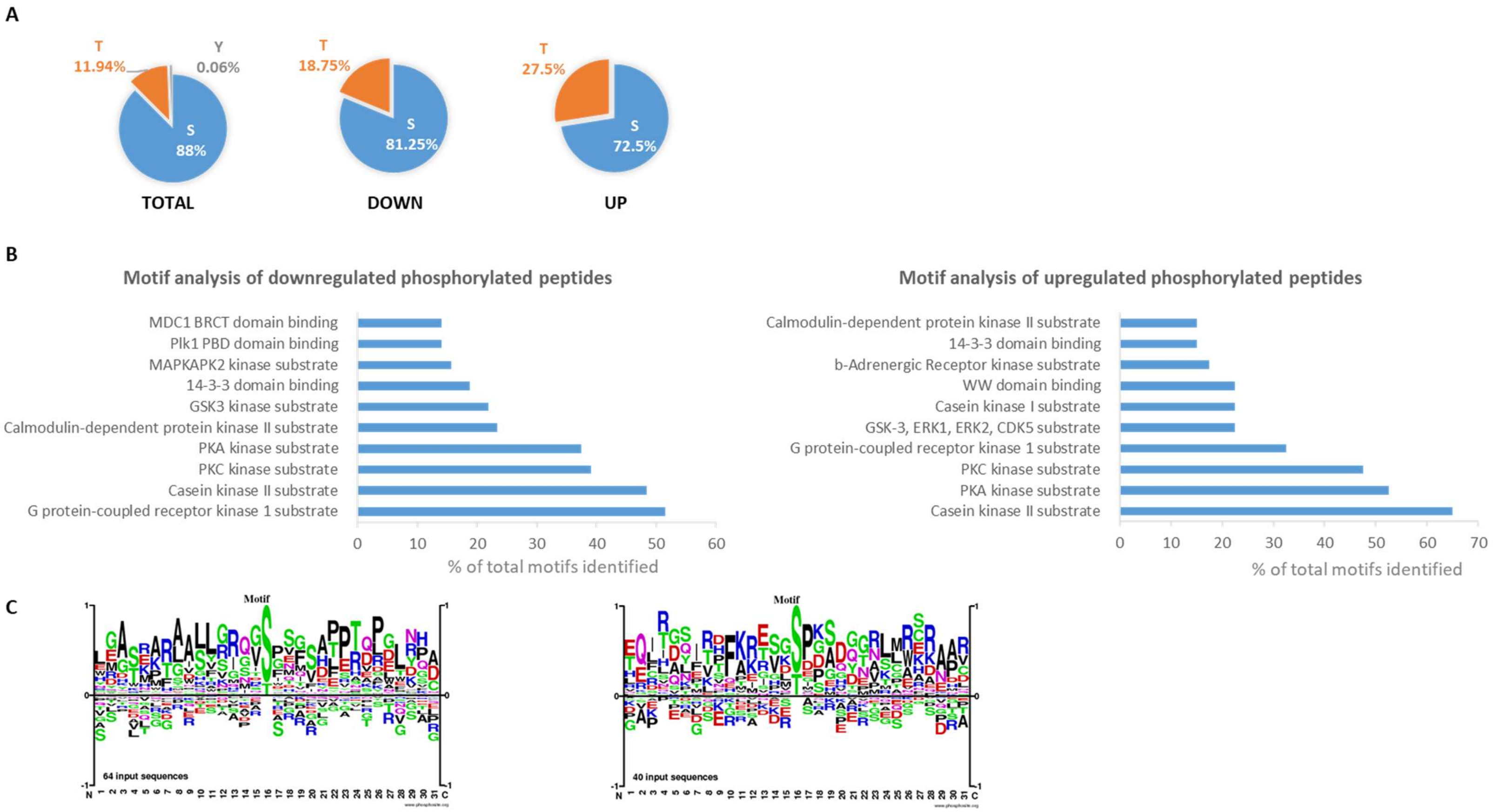

Supplementary Figure 5: Analysis of the phosphoproteome after 2h treatment. A. Proportional representation of phosphorylation sites on serine, threonine and tyrosine for total phosphorylation sites (TOTAL), significant downregulated phosphorylation sites (DOWN) and significant upregulated phosphorylation sites (UP). B. Bioinformatics analysis of potential kinases and/or binding partners based on the surrounding sequence of altered phosphorylation sites revealed the 10 most common kinase and/or binding partner substrate motifs detected among significant phosphoproteins. C. Sequence logos for significant phosphorylation motifs (left for downregulated phosphorylation sites, right for upregulated phosphorylation sites) where the phosphorylated residue (S or T) is centered.
