# Supplementary material for: PP1 phosphatase controls both daughter cell formation and amylopectin levels in Toxoplasma gondii
Source: PLoS Biol. 2024 Sep 10;22(9):e3002791. doi: 10.1371/journal.pbio.3002791 (PMC11414933; doi:10.1371/journal.pbio.3002791)
Supplement: Attachment — Submitted filename: Reviewed by Sarah Reviewer response R1 PP1 - 160624.docx [file pbio.3002791.s013.docx]

Reviewer #1: In this manuscript, the authors investigate the role of PP1 phosphatase during the lytic cycle of Toxoplasma gondii. Using the Auxin Inducible Degron, the enzyme was previously shown to regulate Ca2+ uptake to promote parasite motility (PMID35976251). Here using a similar strategy, the authors identify different requirements for PP1 during the lytic cycle including for the formation of the inner membrane complex, cell division and amylopectin levels. They further investigate the role of PP1 by determining the transcriptome, proteome and phosphoproteomes at two time points following invasion. While little differences are observed at the transcriptome and proteome levels, significant changes are observed in the mutant phosphoproteome. This includes higher abundance of 40 phosphopeptides, some of them mapping on IMC proteins, and 2 phosphopeptides mapping on a putative multidomain trehalose phosphatase. Phosphomimetic or phosphonull mutants did not show a phenotype

in normal culture conditions but showed a correlation between putative phosphorylation of this protein with the presence of Periodic Acid Shiff (PAS) positive vacuoles at pH8.2, a growth condition that induces bradyzoite differentiation.

Major comments

Introduction - The generalization from Toxoplasma to Apicomplexa leads to inaccurate statements. I would strongly suggest the authors to generally use Toxoplasma instead of Apicomplexa unless for specific comparisons.

We agree with the reviewer and focused on Toxoplasma in the introduction.

There is no formal analysis or comparison with the phenotypes observed here and the phenotypes previously described in PMID35976251. These phenotypes do not overlap and look at time discrepant. For example, the IMC defects were either missed or absent in the previous study that only referred to delayed intracellular growth. While I do not want to question the validity of the results of one or the other study, it would be important to clearly address this point to infer why the described phenotypes are different and somehow discrepant.

In PMID:35976251, the authors focused on egress and motility of the mutant parasites. The authors examined the phenotypes of parasites treated with auxin a few hours (4 hours) before egress, at a time where intracellular growth is not anymore impacted by the depletion of PP1 and when PP1 is re-localized to the apical pole of the parasite. None of these phenotypes was assessed in our study as we focused on cell-cycle dependent defects. However, the authored noted the absence of plaque formation, similar to what we observed (Figure 1). We therefore think that both studies are complementary by examining different aspects of the tachyzoite cycle and that the phenotype we observed are not different or discrepant. This is clearly stated in the discussion (lines 594-604)

It would be important to better describe the proteome results and to get an idea about their depth. This also holds true for the phosphoproteome with no GO term enrichment nor motif analysis. This would help to get a better idea of the global changes. Of note many proteins highlighted are not IMC proteins and the authors should be careful and more comprehensive in their description of the dataset.

In the revised version of the manuscript, we provide a more complete analysis of the phospho-proteomics dataset. Supplementary figures 5 and 6 present the motif analysis and GO enrichment. Of note, very few GO pathways were significantly enriched. This is mainly due to the lack of annotation of a lot of genes in the *T. gondii* genomes, even the ones that were identified as IMC proteins in published studies. We also changed the figure 5 legend to reflect the fact that there were more than just IMC proteins in the dataset.

Could the authors discuss the possible reasons explaining the higher abundance of phosphopeptides mapping on IMC protein? Would this be due to mislocalization of these proteins in the absence of IMC? Or is the defective IMC formation due to trafficking defect for example? The results presented here are correlative but give limited insights in explaining the cause of the observed defects.

The higher abundance of phosphopeptides of IMC proetins is not due to protein mislocalization (figure 5E) or higher abundance of the protein (Figure 5F and supp figure 4B). Rather, as we discussed in lines 618-631, the possible effects of phosphorylation would generally promote disassembly of the protein network formed on the IMC as it has been showed for the nuclear lamina network.

Similarly, the link between PP1 and the putative trehalose phosphatase is currently difficult to establish in absence of direct comparisons with PP1 under similar experimental conditions (pH of the culture in this case).

The PP1 mutant accumulated amylopectin in absence of pH-stress much like what has been shown for CDPK2 or PP2A mutants. However, the effect of the trehalose phosphatase phosphosite mutants was more evident when under alkaline stress. We think that we could not recapitulate with single phosphomutant the extent of the perturbations that are caused by the lack of PP1, even in absence of alkaline stress. This is now stated in lines 695-699.

In addition, multiple mutants have been previously associated with altered amylopectin metabolism but with different phenotypes including the localization of storage granules. This is here not shown for the trehalose phosphatase mutants.

We also noted the difference in localization of the amylopectin granules. In our mutant, the AG were mainly cytoplasmic while in the CDPK2 and PP2A mutants, they were both cytoplasmic and in the residual body. We think both pathways are independent (discussed in lines 699-704). The level of amylopectin accumulation may be also more important in the latter although there is no way to directly compare these in the different mutants.

Of note, it is a bit worrying to see different amino acid numbers for the mutated sites across the panels and figure legends.

We apologize for this mistake that has been corrected in the figure.

Finally, while I am not an expert in metabolism, could the authors speculate in details about the link between a trehalose phosphatase and amylopectin accumulation?

Trehalose and trehalose-6-phosphate has been shown to play important regulatory roles in starch accumulation in plants although the molecular mechanisms are not clearly described. This is now discussed in lines 717-726.

Minor comments

line 40-41 - I would use "has been used" instead of "emerged" - in the case of amylopectin, Toxoplasma does not seem to be a model for Plasmodium

This has been changed in the text.

line 59 and after - Phosphorylation is generally regarded as a post-traductional modification not post-transcriptionnal modification even if this is technically correct

This has been changed throughout the text.

line 64 - given the importance of the centrosome in the result section, the specificities of the Toxoplasma centrosome could deserve a bit more attention in the introduction

More context has been added on the centrosome in lines 63-68.

line 381 - I would replace "essential" by "important"

The text has been changed according to the reviewer’s comment.

Fig S3 - please define better how the centrin to nucleus ratio is determined. As a non-expert, I see less centrin dots in the lower panel unless the authors consider the tiny dots as normal centrin staining. In addition, the determination of the number of nuclei numbers seems non-trivial given the "diffuse" DAPI signal - could the authors better describe the criteria used for this quantification?

For each nucleus, the number of Centrin dots is accounted for independently from their size (size and shape varies depending on the cell cycle stage). Overall, more than 100 individual nuclei are counted for each biological replicate. This is now stated in the figure legend to clarify this point.

line 428 - the transition to amylopectin seems to come out of nowhere (as far as I know, investigating amylopectin levels is not a systematic phenotyping in the field of apicomplexan kinases/phosphatases). Are amylopectin granules already visible in the EM images of Figure 2, this could justify more attention?

Amylopectin steady state levels were previously reported to be regulated by phosphorylation of the enzymes of the pathway. This is why we have looked at this phenotype. This is explained in lines 461-462.

line 438 - how do the authors know that the observed structures seen by EM are amylopectin granules?

In the revised version of the manuscript, we performed EM using a specific technique that allows the identification of starch using periodic acid. This is now stated in the Material and m]Methods.

line 481 and elsewhere - I am not sure that the terminology hyper/hypophosphorylated is correct. Does it mean that more or less sites are phosphorylated on the detected peptides? As far as I understand the authors measure the relative abundance of phosphorylated peptides, if I am wrong please define better what hypo/hyperphosphorylated means.

The reviewer is right, we measured the relative abundance of phosphorylated peptides. We define as hypophosphorylated the phosphopeptides that are less abundant after depletion of TgPP1 and as hyperphosphorylated the phosphopetides that are more abundant after depletion of TgPP1. This terminology was also adopted by Herneisen *et al.*

line 569 - please also discuss PMID24779470 that showed that deletion of twelve phosphorylation sites within GAP45 did not impact its dual function in motor recruitment and pellicle integrity. This may give a more balanced view of our exact knowledge on how phosphorylation regulates biological processes in Toxoplasma.

This is now discussed in lines 643-646.

Line 599 - the phenotype described for PP1 seems different from the CDPK2 phenotype, it would be important to discuss these differences

This now discussed in lines 690-695.

Reviewer #2:

The authors have presented their interesting finding on conditional knockdown of PP1 in Toxoplasma, an apicomplexan parasite. The role of PP1 in Toxoplasma has also been investigated in number of studies earlier that authors do mention in their introduction.

Though it is mainly concentrated on Toxoplasma but they completely ignore the various recent studies in closely related apicomplexan Plasmodium falciparum both in their introduction and the discussion with respect of number of function they investigated here (PMID: 32669539, PMID: 34145386; PMID: 35336160; PMID: 33036936; MID: 37628837). It is surprising as the group is working in the same institution where PP1 has been studied widely in Plasmodium by Khalife group. So to complete ignore work in closely related apicomplexan Plasmodium is quite intriguing. They do not review and mention about the similar finding in Plasmodium in terms of IMC and other defects of PP1 knockdown.

We agree with the reviewer that we missed out on some important work on *Plasmodium* PP1. We now discuss and compare the phenotype of mutants that were produced in *P. berghei* or *P. falciparum* lines in 87-91 of the introduction and lines 594-599.

So the novelty and significance of the finding is bit subdued because of these recent findings. Fully appreciate that it has been just been repeated in Toxoplasma. Many of the technical approaches are also similar to what has been done in Plasmodium. The only additional information in terms of function of PP1 in Apicomplexa that is important is the its role in starch production that has not been shown earlier.

We disagree with the reviewer and we think that many aspects of TgPP1 biology were not investigated before our study although we admit that some of the technical approaches are not novel.

It is not clear from the study presented as to what is the focus of the authors to whether they want to analyse the function of PP1 during cell cycle, cell division or the starch metabolism. The different part of the study does not really focus on any one aspect in more detail. It is clear from various studies on PP1 in eukaryotes that PP1 has varied roles but it would have been better if the authors had focussed on any one aspect in more detail here.

The few suggestions to have some clear focus either cell cycle, cell division or starch metabolism with PP1 in Toxoplasma are as:

1. It will be informative if the authors present some good high quality images with either expansion microscopy or the zoom of the confocal images. Please shows the clear defect in the cell division and segregation with some markers like for kinetochore Ndc80, gamma tubulin etc for defect in division and segregation. This has been performed very elegantly in Toxoplasma in many recent studies. This can performed with different phases of cell cycle to get this part bit more informative. It will be interesting to know if the chromosome segregation or the DNA replication both affected. From the images presented in the manuscript it is not very clear about the defect in organelle division.

We performed expansion microscopy to analyze in further details the phenotypes observed in IMC defect and nuclear segregation (Figure 2B). From these images, it appears that rather than DNA replication or chromosome segregation, it is the coordination between the nuclear cycle and the budding cycle that is affected in the mutant. This is further supported by differential phosphorylation after depletion of TgPP1 of TgCep250, a centrosomal protein that has a function in coordinating both cycles. In supplementary figures 3c/d, we also examined mitosis using ExM and markers of the kinetochore (NDC80) and alpha tubulin (cytoskeleton, spindle, centriole and daughter cell cytoskeleton). We detected a delay in exiting metaphase although the parasites seem to eventually finish the nuclear cycle. This further demonstrates that the nuclear segregation defects are due to a default in coordination of the nuclear and budding cycles. We also added Zoom pictures (figure 3b) of the organelle segregation defect observed to better represent the defect that was quantified in figure 3c.

2. Some clear markers for IMC and glideosome component could be studied for as they show that in their phosphoproteome data that these components are affected. Can they look for the component of the IMC complex or glideosome complex that is affected in PP1 knockdown parasite and at what stage during intracellular stage.

As per the reviewer’s request, we performed IFA and ExM on another marker of the IMC (IMC3) replicating the phenotypes (Figure 2a/b) that were observed using IMC1, GAP45 and ISP1 (suppl figure 2). In addition, we performed ExM using IMC17 as marker of the IMC (Figure 5e). Indeed, multiple peptides of this protein are differentially phosphorylated in absence of TgPP1. These data provide further evidence of the IMC phenotype observed in the mutant.

3. For the starch metabolism part- can they please perform lipidomics to show what component of these starch granule are affected.

We did not perfom lipidomics as investing lipids to examine a storage sugar or sugar metabolism was beyond the scope of this study.

Some points on the figures etc:

Fig1 d. It seems at 48h there is still growth. What happens after 48 hours?

The plaque assay indicates that the mutant parasites do not proliferate further after the first cycle. After 48h, the parental strain and the mutant strain without auxin lysed out from cells, and therefore it is difficult to compare growth with these parasites. The accumulation of structural defects in the mutant with auxin also impedes our ability to count the number of parasites per vacuole.

Fig2 How many cells counted. Does not look like DNA has even replicated. Would be great to quantify DNA here before saying missegregation phenotype. Also for EM images how many were counted with he defects and did all showed the same defect. It will be good to look in depth for defect with in the nuclear compartment as well with some markers.

We counted 100 vacuoles for each of the biological replicates (n=3) as stated in the materials and methods. As illustrated by figures 2a and 2b, parasites without nucleus or nucleus without parasite is the overwhelming phenotype (as quantified in figure 2c). ExM s was also used to characterize this phenotype (figure 2c and 5f). Quantification of the IMC defect in EM images is also provided in sup figure 2e.

Fig3. phase images are not clear and may be some zoom of the regions are given or expansion microscopy is performed with these marklers to show clear defect in Fig3a.

As per the reviewer’s request, we produced Zoom images of the normal and abnormal phenotypes for plastid and Golgi segregation. See figure 3b. We also changed the contrast of the images.

Fig4 Again the phase are not clear to understand the cell structure. The quantification EM images will be good. At what stage of cell cycle these images were captured.

We removed the phase images since they were not of satisfying quality. We provided the quantification of EM images below. Although the quantification clearly is in concordance with the other results, we decided not to include it in the manuscript since the phenotype has been evaluated by two different methods by more statistically robust methods.

Figure 1: Quantification of the number of amylopectin granules per parasites. The mean number of amylopectin granule per parasite is plotted for the iKD TgPP1 parasite without (n=20) or with auxin treatment for 24h (n=19) or 48h (n=24). A t-test was performed. *: p<0,001.

Fig5. Labelling are too small to see the protein names and axes.

We increased the size of the font in the figure.

Reviewer #3: This manuscript from Khelifa and colleagues describes multiple cellular functions of an essential phosphatase in the eukaryotic pathogen Toxoplasma gondii. They have engineered an inducible knockdown parasite line to evaluate the role of TgPP1 during tachyzoite replication. Using a phosphoproteomics approach, they have identified a number of PP1 targets involved in many cellular processes, including regulation of amylopectin levels. As accumulation of amylopectin is a hallmark of Toxoplasma chronic stage infection and little is known about how the parasite regulates this metabolic process between acute and chronic stages, identification of regulators like PP1 and its target TGME49_297720 will be of interest to the field. The experiments have been performed and analyzed appropriately, the data has been well presented (apart from a few small errors noted below) and the conclusions made by the authors are supported by the reported results.

This is not the first report of an inducible PP1 knockdown in Toxoplasma tachyzoites, so although the impact is somewhat diminished, this study does yield novel findings on the role of this phosphatase in organelle division and segregation, parasite structure and amylopectin metabolism.

Major comments

I recommend that the authors consider removing "control of tachyzoite cell cycle" from the title of the paper. There is no evidence presented in the manuscript that PP1 directly regulates cell cycle progression. Perhaps something like "The PP1 phosphatase exhibits pleiotropic roles controlling both daughter cell formation and amylopectin-steady state levels in Toxoplasma gondii" is a little more representative of the data in the paper?

We changed the title according to the reviewer’s comment.

Figure 1d. An average of four parasites per vacuole after 48hr of growth for both parental and iKD TgPP1 parasites, with or without auxin. This is very low. Does this represent parasites that have egressed from the first round of intracellular replication and reinvaded? Or have the parasites been pretreated with auxin for a period, and then inoculated into plates with coverslips for further culturing up to 48hr of total treatment time? A more detailed description of the experimental set up of this growth assay is needed to clarify this.

The previous version of the figure was indeed incorrect. We apologized for this error. We proceeded to recount the growth assay at 48 hours and the data presented in the new version of the figure is correct and shows an average number of parasite per vacuole above 16 parasites at 48 hours. This number is influenced by some egress and reinvasion in the parental parasites yielding a higher number of vacuole of 1 parasite in this strain. Although, it is difficult to avoid this technical problem, we think that the figure is representative of the growth defect observed in the iKD TgPP1 strain.

Another recent paper (ref 14, Herneisen et al) reported on phenotypes of Toxoplasma tachyzoites during PP1 inducible knockdown and included a phosphoproteomics approach like the one reported here. Although this paper has been referenced a couple of times in this manuscript, a more thorough discussion of the important differences between the two studies should be included. Did the phosphorylation marks identified in both studies display the same trend i.e. increase or decrease during PP1 knockdown? There are some important technical differences in the experimental approaches used in these studies (differences in timing, perturbing calcium levels etc.) so it would be relevant and helpful to interested readers to highlight what is different with this study and what has been learned from this approach over the previous one.

We agree with the reviewer and included a more detailed comparison of the two datasets. See lines 553-558 for the phosphoproteomics and lines 594-604

for the phenotypes. We want to emphasize that comparison of the proteomics datasets is strongly diminished by the technical differences. For example, we performed at least three biological replicates (two in Herneisen *et al.*) and a different statistical test than Herneisen et al. Moreover, the parasites were harvested at different time points and with different duration of auxin treatment.

Minor comments

Line 23 eukaryotic, not eucaryotic

The text was changed according to the reviewer’s comment.

Line 47 consumption not consummation

The text was changed according to the reviewer’s comment.

Line 72 it would be more precise to say that homologues of TgPP1 have been studied in other organisms, rather than TgPP1 is a phosphatase that has been studied in other organisms.

The text was changed according to the reviewer’s comment.

Line 81-83 The reference for interaction of PP1 with LRR1 (Daher et al 2006) refers to a paper on Plasmodium falciparum. There is no experimental evidence for interaction between these two protein homologues in Toxoplasma.

The reviewer is right, we changed this reference to Daher W et al. 2007 (PMID: 17660360).

Line 482 should say "hyperphosphorylated" not "hypophosphorylated"?

The text was changed according to the reviewer’s comment.

Line 523 refers to supplementary figure 5, but a supplementary figure 5 has not been provided.

Supplementary figure 5 (now supplementary figure 7) is now provided.

Figure 6b. Labels on the IFAs have the mutated amino acids as 1053 and 1078, should be 1054 and 1073

The figure was changed according to the reviewer’s comment.

COMMENTS FROM THE ACADEMIC EDITOR:

Line 396 misegregation of nuclei and fig2a,

parental and kd dapi stain look the same in fig 2a. is dapi stain the measure of nuclear segregation?

We clarify what we call nuclear segregation defect inLines 420-421. We also provide a clearer picture of the phenotype by the use of expansion microscopy (Figure 2b).

line 418 centrosome shape suppl fig 3a

this is not convincing on the basis of one image and both parental and pp1 kd have elongated centrosomes

We agree with the academic editor and removed the statement.

fig 3b labelling needs to be improved

We improved the labelling of the bar graph of figure 3b (now 3c) by changing the font size.
