# Supplementary material for: PP1 phosphatase controls both daughter cell formation and amylopectin levels in Toxoplasma gondii
Source: PLoS Biol. 2024 Sep 10;22(9):e3002791. doi: 10.1371/journal.pbio.3002791 (PMC11414933; doi:10.1371/journal.pbio.3002791)
Supplement: Attachment — Submitted filename: Reviewer response.docx [file pbio.3002791.s014.docx]

Reviewer #2:

The authors have addressed many of the comments from the previous version and it reads more focussed. The explanation of the differences to other studies published on PP1 in Toxoplasma is helpful. They have also improved the images adding expansion images and better labelling etc is provided. However I am bit confused with the previous study that they studied the role of PP1 in motility and egress whereas the phenotype here shows abnormal division, so did the author tried to study the PP1kd parasite for the e same phenotype as the previous study reported. Some discussion on it will be helpful. It is also not clear if the IMC and centrosome dynamics are linked during cell division so why PP1 deletion affects only the IMC and not the centrosome division.

We further discussed the possible link between the IMC and the centromal modification caused by TgPP1 depletion.

I have few minor comments :

1. The method section do not describe about the technique of expansion microscopy they have included in the revised version

2. Line 101 may be add chromosome segregation rather than just segregation

3. line 457 it should be "undergoing" instead of undoing mitosis

We made all the changes requested.
